# IL-17A-mediated mitochondrial dysfunction induces pyroptosis in colorectal cancer cells and promotes CD8 + T-cell tumour infiltration

**DOI:** 10.1186/s12967-023-04187-3

**Published:** 2023-05-21

**Authors:** Wen-Qing Feng, Yu-Chen Zhang, Zhuo-Qing Xu, Su-Yue Yu, Jian-ting Huo, Abudumaimaitijiang Tuersun, Min-Hua Zheng, Jing-Kun Zhao, Ya-Ping Zong, Ai-Guo Lu

**Affiliations:** Department of General Surgery, Shanghai Minimally Invasive Surgery Center, Ruijin Hospital, Shanghai Jiao Tong University School of Medicine, Shanghai, 200020 People’s Republic of China

**Keywords:** IL-17A, Colorectal cancer, Mitochondrial dysfunction, Pyroptosis, CD8 + T

## Abstract

**Background:**

Interleukin-17A (IL-17A), a proinflammatory cytokine primarily secreted by Th17 cells, γδT cells and natural killer T (NKT) cells, performs essential roles in the microenvironment of certain inflammation-related tumours by regulating cancer growth and tumour elimination proved in previous literature. In this study, the mechanism of IL-17A that induces mitochondrial dysfunction promoted pyroptosis has been explored in colorectal cancer cells.

**Method:**

The records of 78 patients diagnosed with CRC were reviewed via the public database to evaluate clinicopathological parameters and prognosis associations of IL-17A expression. The colorectal cancer cells were treated with IL-17A, and the morphological characteristics of those cells were indicated by scanning electron microscope and transmission electron microscope. After IL-17A treatment, mitochondrial dysfunction was tested by mitochondrial membrane potential (MMP) and reactive oxygen species (ROS). The expression of pyroptosis associated proteins including cleaved caspase-4, cleaved gasdermin-D (GSDMD), IL-1β, receptor activator of nuclear NOD-like receptor family pyrin domain containing 3 (NLRP3), apoptosis-associated speck like protein containing a card (ASC), and factor-kappa B was measured through western blotting.

**Results:**

Positive IL-17A protein expression was observed in CRC compared to the non-tumour tissue. IL-17A expression indicates a better differentiation, earlier stage, and better overall survival in CRC. IL-17A treatment could induce mitochondrial dysfunction and stimulate intracellular reactive oxygen species (ROS) production. Furthermore, IL-17A could promote pyroptosis of colorectal cancer cells and significantly increase the secretion of inflammatory factors. Nevertheless, the pyroptosis induced by IL-17A could be inhibited through the pre-treatment with Mito-TEMPO (a mitochondria-targeted superoxide dismutase mimetic with superoxide and alkyl radical scavenging properties) or Z-LEVD-FMK (caspase-4 inhibitor, fluoromethylketone). Additionally, after being treated with IL-17A, an increasing number of CD8 + T cells showed in mouse-derived allograft colon cancer models.

**Conclusion:**

IL-17A, as a cytokine mainly secreted by γδT cells in the colorectal tumour immune microenvironment, can regulate the tumour microenvironment in multiple ways. IL-17A could induce mitochondrial dysfunction and pyroptosis through the ROS/NLRP3/caspase-4/GSDMD pathway, and promote intracellular ROS accumulation. In addition, IL-17A can promote the secretion of inflammatory factors such as IL-1β、IL-18 and immune antigens, and recruit CD8 + T cells to infiltrate tumours.

**Graphical Abstract:**

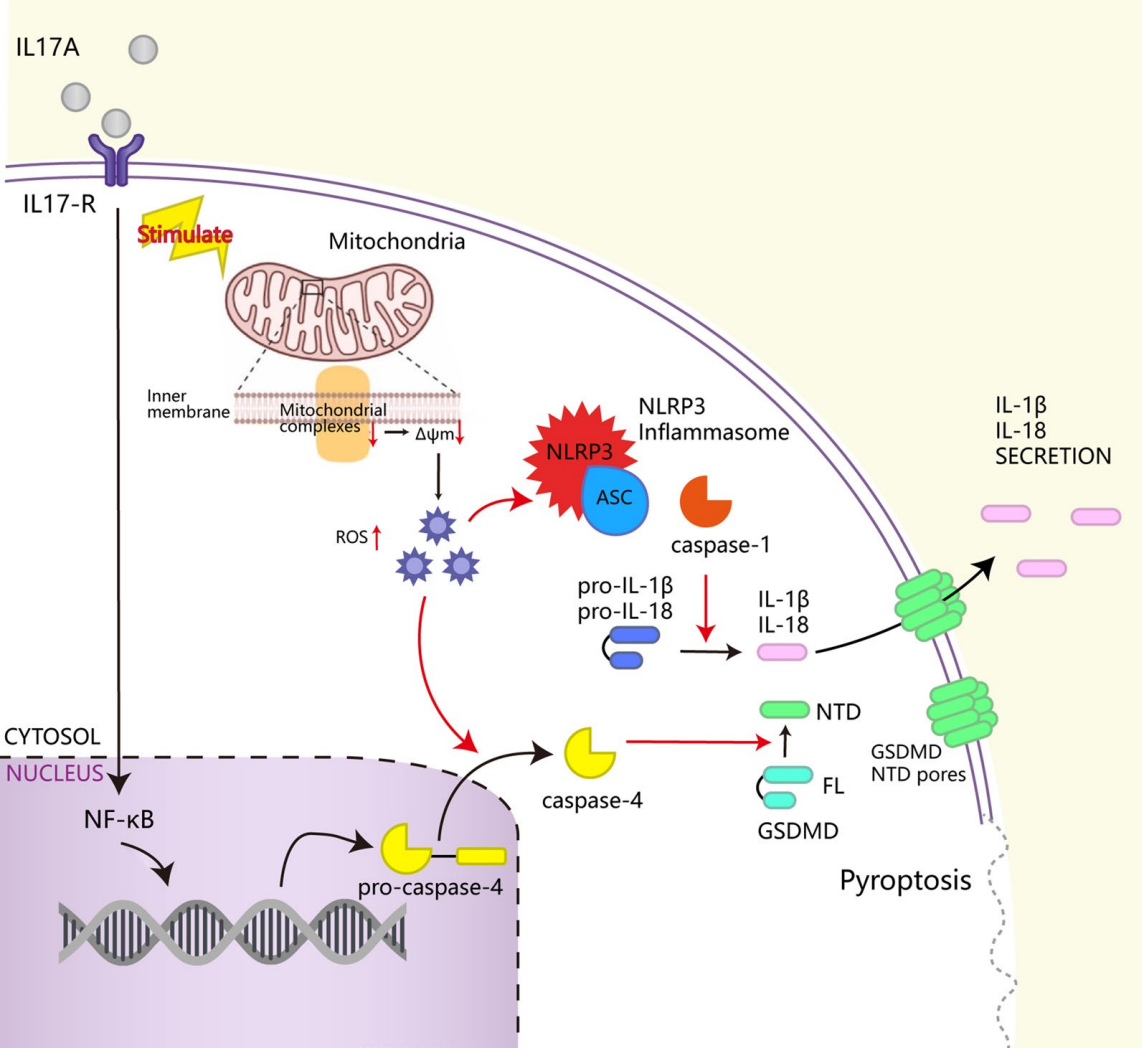

**Supplementary Information:**

The online version contains supplementary material available at 10.1186/s12967-023-04187-3.

## Introduction

Colorectal cancer (CRC) is the third most prevalent and deadly malignant disease in the world [1]. Immunotherapy has changed the treatment outcomes of malignancies that previously carried a dismal prognosis, providing hope for patients with advanced and metastatic lung cancer, renal cancer or melanoma, who may benefit from the remarkable response rates of immune checkpoint blockade [2]. However, the lack of expression of immune antigens and infiltration of T cells has been disappointing in colorectal cancer, in which the relatively immune-cold microenvironment precludes immunotherapy success [3]. Therefore, it is necessary to obtain a deeper understanding of the biochemical microenvironment associated with antitumour immunity in colorectal cancer.

Interleukin-17A (IL-17A), an important proinflammatory cytokine that is mainly secreted by Th17 cells, γδT cells and natural killer T (NKT) cells [4], has been described as a common inflammatory factor in numerous tumour microenvironments, in which it can play dichotomous roles in both tumour growth and tumour elimination [5]. IL-17A promotes cancer growth and progression by directly stimulating cancer cells via activation and translocation of NF-κB, STAT, and AP-1 and signalling that leads to the downstream activation of kinases, such as MAPK and HER1[6]. Similarly, IL-17A has tumour-promoting effects mediated by promoting cancer epithelial-to-mesenchymal transition in vivo via tissue-remodelling matrix metalloproteinases (MMPs), such as MMP-2, MMP-7,and MMP-9 [7]. The expression of IL-17 increases in the premalignant stage of CRC and directly correlates with dysplasia in the colonic adenoma-to-carcinoma sequence, making IL-17 a potential biomarker for colon cancer diagnosis and severity [8].

IL-17A expression correlates with a better prognosis and improved patient survival in a variety of cancers [9], suggesting that IL-17 may have antitumour effects. In both colorectal cancer and lung cancer, IL-17A can recruit antitumour neutrophils, stimulating a T-cell response in the tumour immune microenvironment, which correlates with better overall survival [10, 11]. A case report described a male patient with metastatic colon cancer who had a severe flare-up of previously mild psoriasis after treatment with pembrolizumab (AntiPD1). Unfortunately, progression of colon cancer occurred after anti-IL-17 treatment was given to treat the psoriasis flare [12], supporting a role for IL-17A in antitumour immunity.

In this study, we show that IL-17A is mainly secreted by γδT cells in the immune microenvironment of colorectal tumours. IL17-A promotes intracellular ROS accumulation by inducing mitochondrial dysfunction while inducing pyroptosis by activating the ROS/NLRP3/capsase4/GSDMD pathway, upregulating the release of inflammatory factors such as IL-1β, IL-18 and immune antigens into the tumour microenvironment, and recruiting infiltrating CD8 + T cells to the tumour.

## Methods and materials

### Human samples

Human tissue specimens were obtained from the Ruijin Hospital of Shanghai City, People’s Republic of China. This study was approved by the Institutional Review Board of Ruijin Hospital Ethics Committee (Shanghai Jiao Tong University School of Medicine). Patients who received preoperative treatment (such as radiotherapy or chemotherapy) were excluded from the study. According to ethical guidelines obtained the informed consents written by the patients and anonymously coded all the human tissue samples. All procedures comply with the ethical principles for medical research involving human subjects as set out in the World Medical Association's Declaration of Helsinki (revised 2008).

### Immunohistochemical score

Two independent pathologists scored the intensity of immunohistochemical staining of il-17A and IL-1β in tumor tissues according to a semi-quantitative immunoreactivity scoring system. The percentage of immunoreactive cells was scored as follows: 0% (0), 1–10% (1), 11–50% (2), 51–80% (3) and > 80% (4). The staining intensity was scored as follows: no staining (0), weak staining (1), moderate staining (2) and intense staining (3). These values were multiplied together to provide a single score ranging between 0 to 12 for each case.

### Animal models

Four-weeks-old male BALB/c mice were purchased from the Shanghai Institute of Zoology, Chinese Academy of Sciences. For all experiments involving mice were complied with NIH guidelines and were approved by the Institutional Review Board of Ruijin Hospital (Shanghai Jiao Tong University School of Medicine) Ethics Committee.

The tumour cell line CT26, derived from a mouse colon adenocarcinoma, was inoculated subcutaneously into mice to develop implanted tumours. Histologically, a tumour tissue block of approximately 2 mm^3^ was then harvested and implanted into the colon wall of mice to establish a model mimicking human colon cancer. Each group of mice was allowed to eat and drink freely after surgery.

### Cell culture and cytokine treatment

The human colorectal cancer cell lines SW620 and HT29 were purchased from ATCC and stored at the Shanghai Institute of Gastrointestinal Surgery. Both SW620 and HT29 cells were cultured in RPMI-1640 medium (Meilunbio, China) supplemented with 10% neonatal bovine serum (NBS; Gibco, USA). The temperature of the cell incubator was controlled at 37 °C with a 5% CO2 environment.

### Cell counting kit-8 (CCK-8)

Cell viability was examined using a CCK-8 assay (Sevenbio). Cells were seeded in 96-well plates at a density of 4 × 10^3^ cells per well in 200 μl medium for 24, 48 and 72 h. The absorbance was detected at 450 nm after the cells were treated with 10% CCK-8 at 37 °C for 2 h. Cell viability was calculated as a ratio of optical density values of drug-treated samples to those of controls.

### siRNA silencing of IL-17RA expression

To knock down the expression of IL-17RA, cells were transfected with siRNA oligonucleotides targeting IL-17-RA or a nontargeted control siRNA oligonucleotide (GenePharma,CHINA) under standard tissue culture conditions at 37℃ for 7 h, and then fresh culture medium was added for an additional 24 h. Then, Western blotting was used to evaluate the transfection efficiency.

**Table:** 

Si-IL-17RA-1 (5’ → 3’)	sense:GGAACGAAUCUACCCAUUATT antisense:UAAUGGGUAGAUUCGUUCCAC
Si-IL-17RA-2 (5’ → 3’)	sense:GGUCUGGUUAUCGUCUAUCTT antisense:GAUAGACGAUAACCAGACCGC
Si-IL-17RA-3 (5’ → 3’)	sense:CGUUCAUUCAGCAUUUAUUTT antisense:AAUAAAUGCUAAUGAACGAA
Negative control (5’ → 3’)	sense:UUCUCCGAACGUGUCACGUTT antisense:ACGUGACACGUUCGGAGAATT

### Western blotting and antibodies

Total protein was extracted from cell lysates. Western blot analysis was performed using standard methods [13]. Primary antibodies: anti-NLRP3 (A12694; ABclonal, China);anti-IL17A (A0688; ABclonal, China);anti-IL17-RA (A10052; ABclonal, China);anti-IL1β(A12688; ABclonal, China); anti-IL18(A1115; ABclonal, China); anti-oxidative phosphorylation (OxPhos) complexes (I-NDUFB8, A19732; II-SDHB, A1809; III-UQCRC2, A4366; IV-MTCO2, A11522 and ATP5J, A3751; ABclonal, China).

### Mitochondrial membrane potential (MMP, ΔΨm) measurement

The mitochondrial membrane potential (ΔΨm) through was measured using a mitochondrial membrane potential assay kit (Beyotime, China). When the ΔΨm is high, JC-1 aggregates in the mitochondrial matrix to form a polymer and produces red fluorescence; when the ΔΨm is low, JC-1 is monomeric and produces green fluorescence. Therefore, after JC-1 staining, cells were observed by fluorescence microscopy and quantified by flow cytometry.

### ELISA

IL-1β protein levels in the supernatant were measured using an ELISA kit (R&D Systems, Minneapolis, MN, USA. The concentration of IL-1β(in pg/mL) was calculated using linear regression curves derived from the data for a series of IL-1β protein standards provided in the kit, which were averaged over replicate readings.

### Statistical analysis

The shapiro–wilk test was used to analyze whether quantitative variables followed a normal distribution. The difference between groups of normally distributed data was tested by two independent samples t-test, and the difference between groups of non-normally distributed data was tested by the Mann–Whitney U test. Also, Pearson's chi-square test (minimization expected value > 5) and Fisher's exact test (minimization expected value ≤ 5) were used to tell the difference between categorical data. All statistics were statistically significant when P < 0.05. For long-term outcomes, Kaplan–Meier curves were plotted and patients with positive and negative IL-17A expression were compared using the Log rank test.

All statistical analyses were performed by GraphPad Prism 9.0 software, SPSS26 software and R 4.2.0 software.

### Data availability

The data generated in this study are publicly available in Gene Expression Omnibus (GEO) at GSE127757.

## Results

### An IL-17A expression signature in colorectal cancer is associated with a drastic decrease in overall survival

**Fig. 1:**
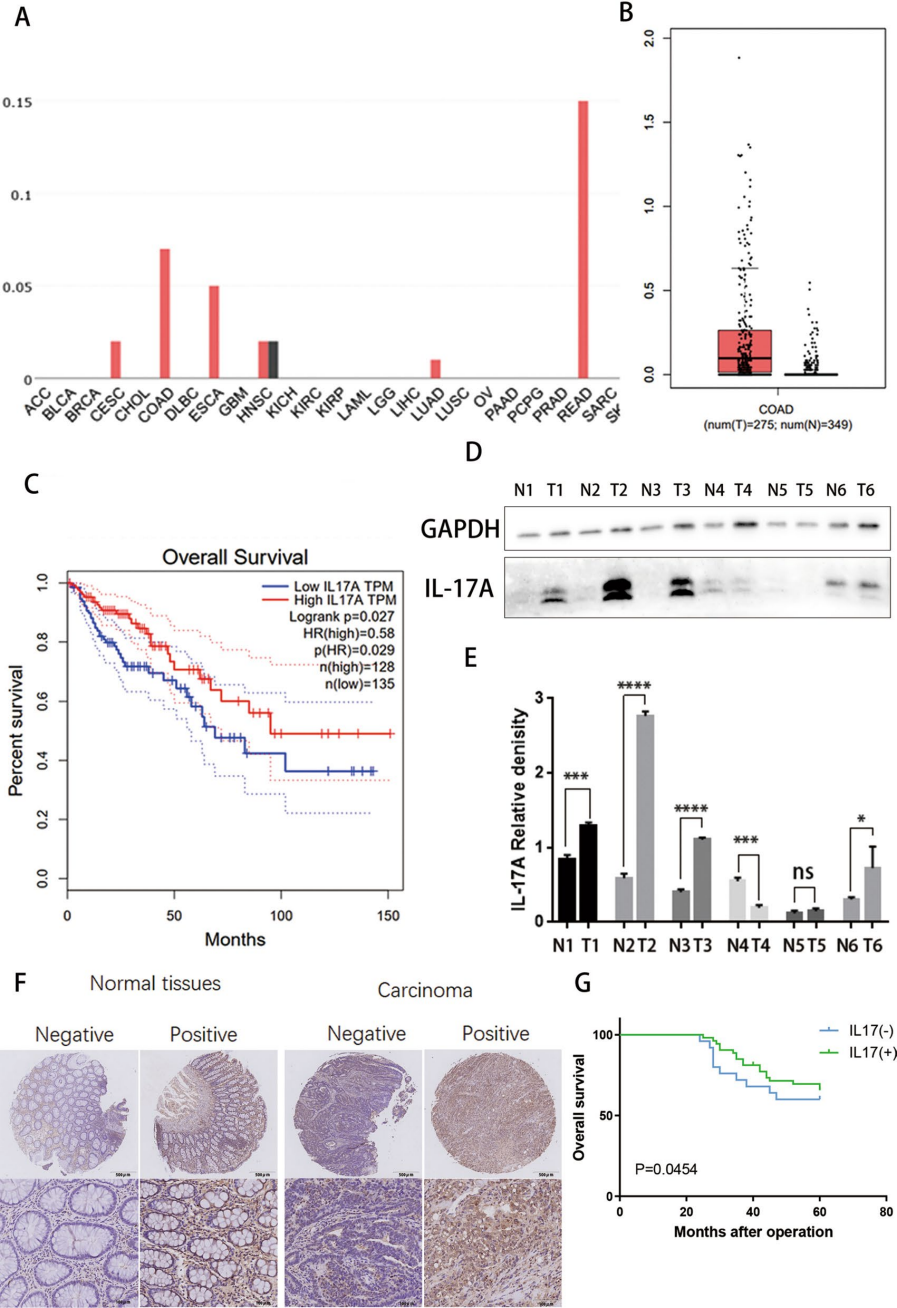
Increased expression levels of IL-17A in patients with CRC. **A** Differentially expressed IL-17A between tumour and normal tissues; ACC: Adrenocortical carcinoma; BLCA: Bladder Urothelial Carcinoma; BRCA: Breast invasive carcinoma; CESC: Cervical squamous cell carcinoma and endocervical adenocarcinoma; CHOL: Cholangiocarcinoma; COAD: Colon adenocarcinoma; DLBC: Lymphoid Neoplasm Diffuse Large B-cell Lymphoma; ESCA: Esophageal carcinoma; GBM: Glioblastoma multiforme; HNSC: Head and Neck squamous cell carcinoma; KICH: Kidney Chromophobe; KIRC: Kidney renal clear cell carcinoma; KIRP: Kidney renal papillary cell carcinoma; LAML: Acute Myeloid Leukemia; LGG: Brain Lower Grade Glioma; LIHC: Liver hepatocellular carcinoma; LUAD: Lung adenocarcinoma; LUSC: Lung squamous cell carcinoma; OV: Ovarian serous cystadenocarcinoma; PAAD: Pancreatic adenocarcinoma; PCPG: Pheochromocytoma and Paraganglioma; PRAD: Prostate adenocarcinoma; READ: Rectum adenocarcinoma; SARC: Sarcomav; SKCM: Skin Cutaneous. Melanoma **B** Differentially expressed IL-17A between colorectal tumour and normal tissues; **C** Comparison of overall survival in IL-17A high and IL-17A low groups; **D**-**E** Representative western blot and quantification analysis of IL-17A expression in paired CRC samples. Data conforms to normal distribution, and tested by two independent samples t-test; **F** Immunohistochemical results showing expression of IL-17A in colorectal tumour and normal tissues; **G** Comparison of overall survival in IL-17A high and IL-17A low groups

We investigated the expression of IL-17A (tumour tissue vs. normal tissue) in the GEPIA (http://gepia.cancer-pku.cn/) database. The results showed that IL-17A was notably upregulated in colorectal adenocarcinomatumour(COAD) tissue compared with paired normal tissue (Fig. 1A&B). To further substantiate the results, we randomly selected six paired samples from colorectal cancer patients and performed Western blot analysis of IL-17A expression. We found that IL-17A levels were higher in colon adenocarcinoma tissue and almost undetectable in normal colon tissue (Fig. 1D and E). Further Kaplan‒Meier analysis of colon adenocarcinoma(COAD) data in the GEPIA database indicated relatively high expression of IL-17A in CRC patients with better survival (p = 0.027 < 0.05) (Fig. 1C).

A tissue microarray (TMA) containing 78 pairs of cancerous and matched noncancerous tissues was analysed using immunochemistry. According to the immunohistochemical staining results, when both CRC tumour tissue and paired normal tissue were scored, a score no lower than 6 was regarded as positive (scoring criteria see materials and methods) (Fig. 1F). Compared with normal tissue, positive expression was more frequent in carcinoma tissue. Then, we further explored the relationships between the expression of IL-17A and the clinicopathological characteristics of CRC patients. The results showed that positive IL-17A expression correlated with better histological differentiation and earlier TNM staging (Table 1). Further Kaplan‒Meier analysis indicated positive expression of IL-17A in CRC patients with better overall survival (p = 0.0454 < 0.05) (Fig. 1G).

**Table 1:** Patient characteristics

Variable	Cases(n)	IL17 expression		P-value
		Negative	Positive	
Tissues				
Carcinoma	78	25	53	0.0012
Normal tissues	78	46	32	
Age (years)		61.2 ± 1.7	60.8 ± 2.7	0.8984
Gender				
Male	41	10	31	0.1502
Female	37	15	22	
Tumor size				
≤ 5 cm	56	16	40	0.4189
> 5 cm	22	9	13	
Differentiation				
Well	31	10	21	0.0456
Medium	38	9	29	
Poor	9	6	3	
TNM stage				
I II	41	7	34	0.0037
III VI	37	18	19	
Distant metastasis				
Negative		21	35	0.115
Positive		4	18	

### IL-17A expression is positively correlated with activated CD4 + and CD8 + T-Cell abundances in the CRC samples

**Fig. 2:**
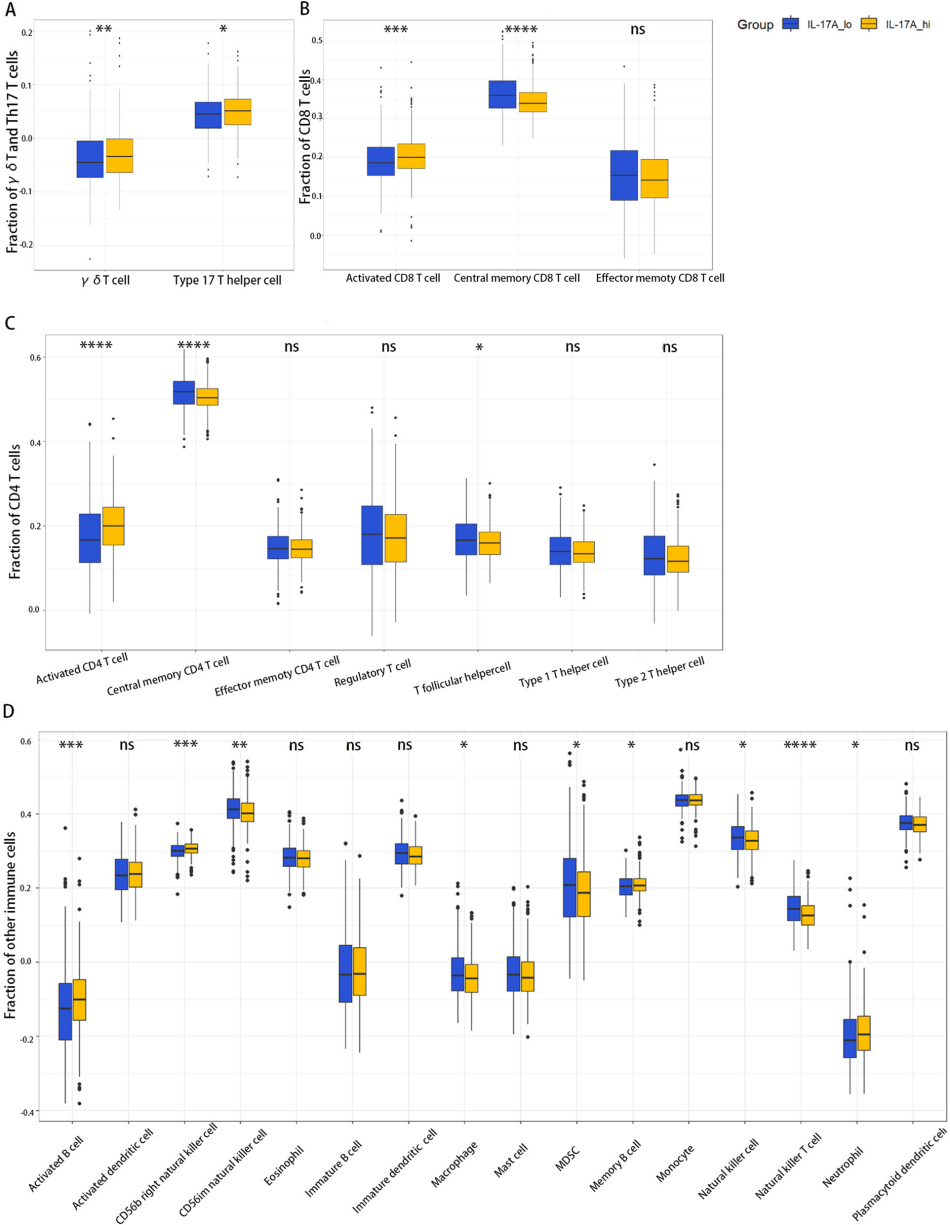
Boxplots showing the relative abundances of immune cell types among the IL17A-hi group and IL17A-lo group. **A**The relative abundances of γδ T cells among the IL17A-hi and IL17A-lo groups; **B** The relative abundances of CD8 + T cells and their subsets among the IL17A-hi and IL17A-lo groups; **C** The relative abundances of CD4 + T cells and their subsets among the IL17A-hi and IL17A-lo groups; **D** The relative proportions of other immune cells among the IL17A-hi and IL17A-lo groups. The difference between groups of normally distributed data was assessed by independent samples t-test, whereas the difference between two groups of non-normally distributed data was assessed by the Mann–Whitney U test. ns, not significant; *P < 0.05; **P < 0.01; ***P < 0.001; ****P < 0.0001

We next explored the relationship between the expression of IL-17A and immune infiltration. All CRC patient data containing complete RNA-seq data were downloaded from The Cancer Genome Atlas (TCGA). A total of 537 tumour tissue samples were divided into IL17A-hi and IL17A-lo groups (247 patients in each group) based on the median geometric mean expression value. ssGSEA (single sample Gene Set Enrichment Analysis) was used to investigate differential immune cell infiltration. γδT and Th17 cells are the two inflammatory cell types primarily responsible for the production of IL-17A in the tumour microenvironment, and the results display that the γδT cells (p = 0.0038 < 0.01) and Th17 cell (p = 0.0213 < 0.05) abundances in the IL17A-hi group were significantly higher(Fig. 2A). In addition, both the activated CD4 + (p = 2.6e-07 < 0.0001) and CD8 + T-cell (p = 0.0004 < 0.001) abundances in the IL17A-hi group were significantly higher (Fig. 2B, C). Furthermore, the results for other immune cell proportions showed that the relative abundances of activated B cells (p = 0.0007 < 0.001), CD56b right natural killer (NK) cells (p = 0.0003 < 0.001) and neutrophils (p = 0.029 < 0.05), were increased and those of macrophages, natural killer T (NKT) cells (p = 1.09e-06 < 0.0001) and MDSCs (p = 0.046 < 0.05) were decreased in the IL17A-hi group (Fig. 2D).

### IL-17A treatment regulates the expression of mRNA in colorectal cancer cells

To investigate the biological function of IL-17A, we screened for the highest IL-17R expression and selected the colorectal cancer cell line SW620 (Additional file 1: Fig. S1A). SW620 cells were treated with 0, 5, 50, 100 ng/mL IL-17A for 24 h, 48 h and 72 h, to identify how different concentrations of IL-17A affected cell proliferation (Fig. Additional file 1: S1B). There was no discernible difference in proliferation after 24 or 48 h of IL-17A stimulation, but at 72 h of treatment, 100 ng/mL IL-17A had an inhibitory effect on proliferation (Additional file 1: Fig. S1C). To investigate the effect of IL-17A on colorectal cancer cells, we observed the morphology of the cells with a microscope. When SW620 cells were stimulated with 100 ng/ml of IL-17A for 24, 48and 72 h, cell swelling and large bubbles were observed across the membrane surface of the osteoblasts after 3 days, which were identified as the characterization of pyroptosis (Additional file 1: Fig. S1D). Therefore, we treated CRC cells with 100 ng/mL IL-17A for 72 h in all subsequent experiments.

**Fig. 3:**
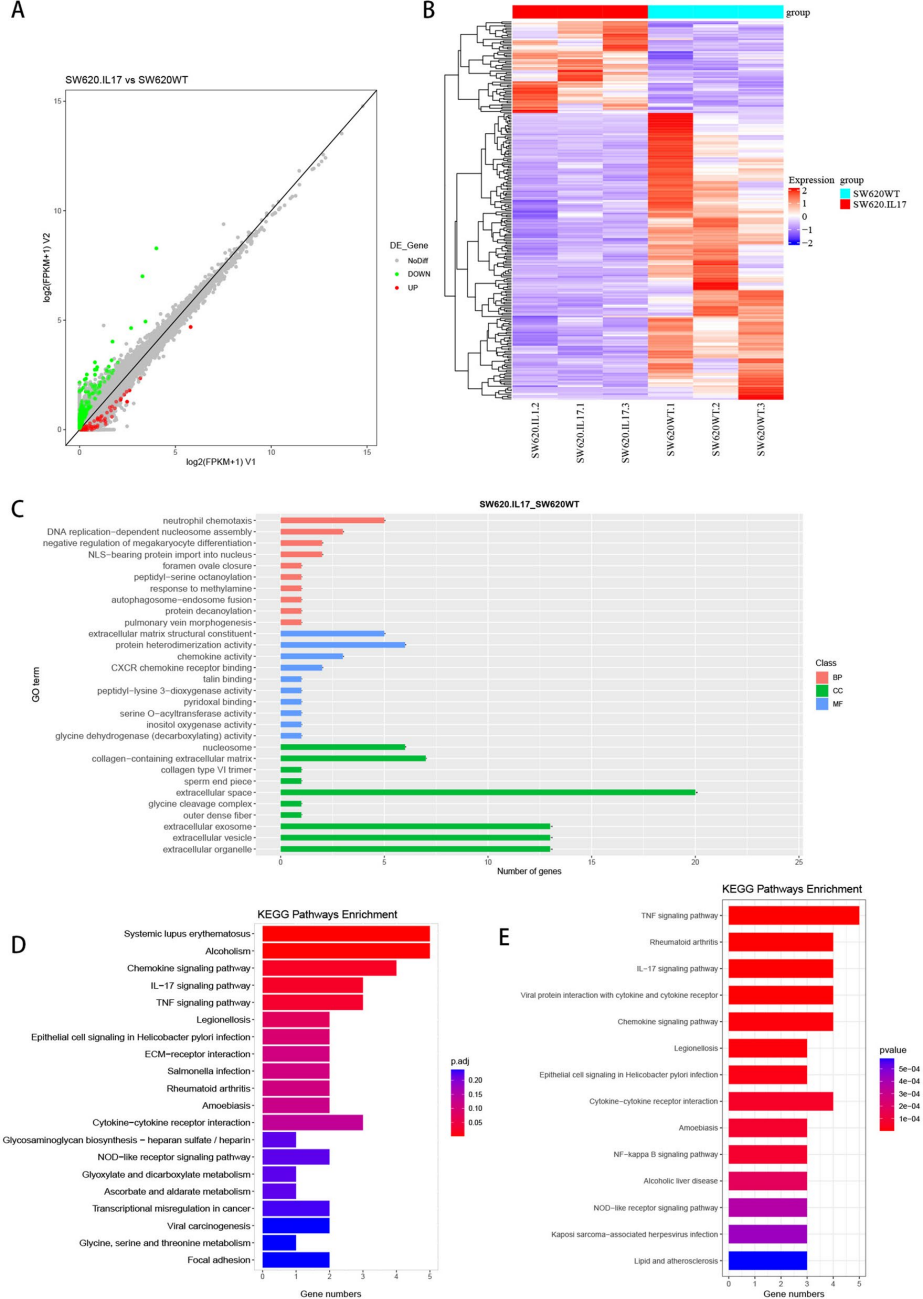
IL-17A activates NF-kB signaling pathway in CRC. **A**, **B** Differential genes expression of the IL-17A group versus that of the control group(pvalue < 0.05 and FoldChange (FC) ≥ 2); **C** Enrichment of the Gene Ontology by DEGs of the IL-17A group versus that in the control group; **D** Enrichment of the Kyoto Encyclopedia of Genes and Genomes pathway by DEGs of the IL-17A group versus that in the control group; **E** Enrichment of the Kyoto Encyclopedia of Genes and Genomes pathway by DEGs of the GSE127757

We performed unbiased RNA sequencing experiments with total mRNA from SW620 cells treated with or without IL-17A for 72 h. Differential gene expression (DEG) was identified in colorectal cancer cells after IL-17A treatment (Fig. 3 A&B), and Gene Ontology (GO) (Fig. 3C) and Kyoto Encyclopedia of Genes and Genomes (KEGG) enrichment analyses (Fig. 3D) were performed to screen for signalling pathway. We found that in addition to the IL-17A signalling pathway, upregulated DEGs were enriched in pathways highly associated with nuclear transcription and inflammatory factor production and secretion, including activation of the Tumour Necrosis Factor (TNF) signalling pathway and NOD-like receptor signalling pathway, which are both highly associated with nuclear factor kB (NF-kB). We used external data for further validation, searched the Gene Expression Omnibus (GEO) database and found the GSE127757 profile, which is Gene expression data for human normal colon organoids stimulated with IL-17A (Fig. 3E). KEGG analysis of the data showed that the the enriched signalling pathways were similar to those identified in IL-17A-treated SW620 cells, and we believe that the experimental results are authentic and credible.

### IL-17A promotes pyroptosis in colorectal cancer cells

**Fig. 4:**
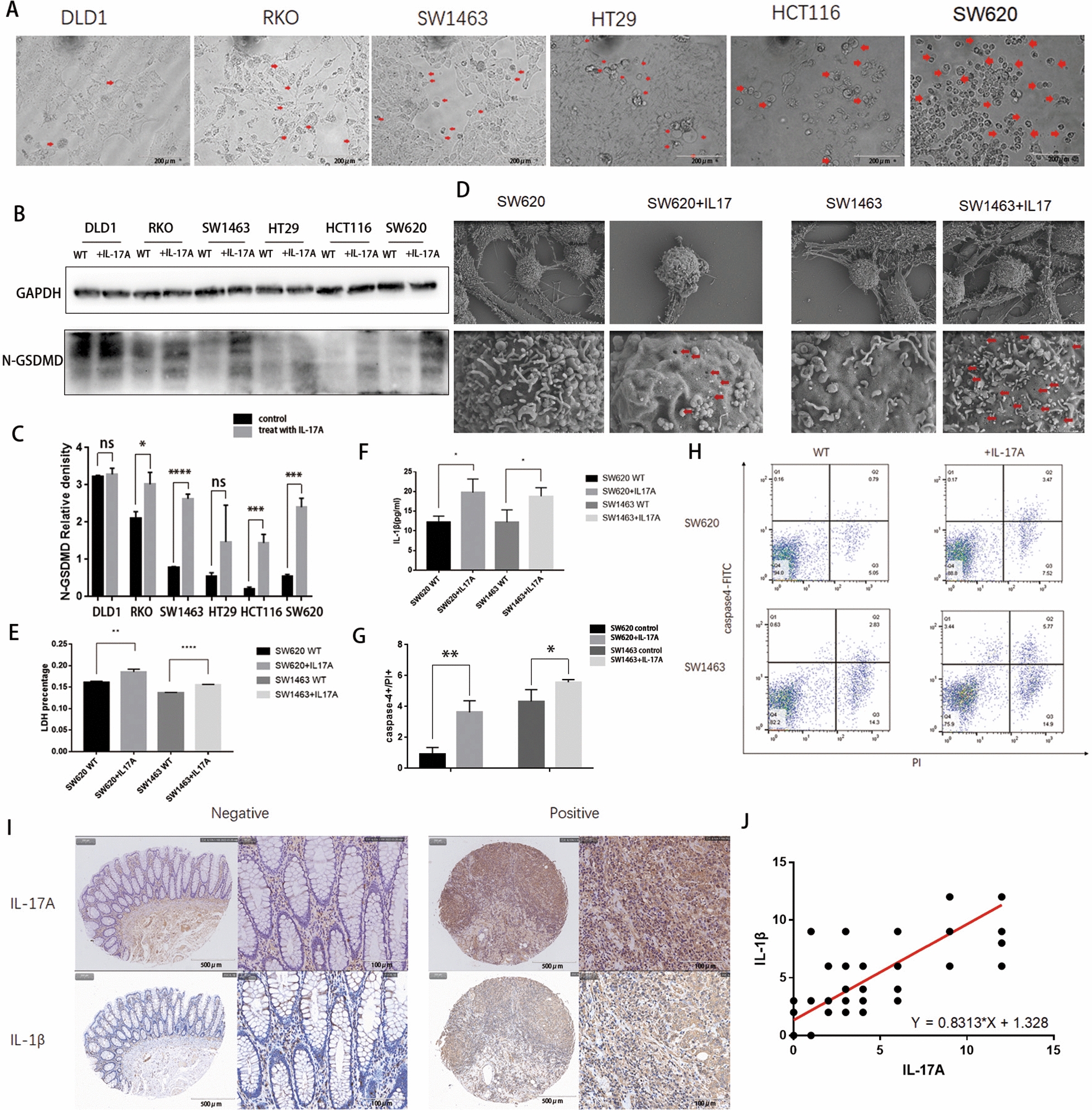
The morphologic and molecular characteristics of IL-17A–induced pyroptosis in CRC cells. **A** Representative microscopic images of CRC cells treated with IL-17A (100 ng/ml) for 72 h. Red arrows indicate the characteristic balloon in the cell membrane; **B**-**C** Expression of GSDME-N terminal by western blotting analysis after treated with IL-17A (100 ng/ml) for 72 h, data conforms to normal distribution, and tested by two independent samples t-test; **D** Representative scanning electron micrographs of CRC cells after treatment with IL-17A.Red arrows indicate formation of pyroptotic membrane pits and pores of varying size; **E** LDH release was detected in the supernates of CRC cells treated with IL-17A (100 ng/ml) for 72 h, data conforms to normal distribution, and tested by two independent samples t-test; **F** The release of IL-1β in the supernate was determined by ELISA, data conforms to normal distribution, and tested by two independent samples t-test; **H**-**G** Cell apoptosis rates were determined by using caspase-4 and propidium iodide co-dyeing staining assay after CRC cells treated with IL-17A (100 ng/ml) for 72 h, data conforms to normal distribution, and tested by two independent samples t-test; **I** Immunohistochemical results showing expression of IL-17A and IL-1β in colorectal tumour and normal tissues **J** Correlations between IL-17A and IL-1β in CRC

To further determine the influence of IL-17A on colorectal cancer cells, DLD1, RKO, SW1463, HT29, HCT116 and SW620 cells were treated with IL17A for 72 h. We observed more or less swelling with large bubbles in the different colon cancer cell lines (Fig. 4A). Moreover, after IL-17A treatment of these cancer cells, N-terminal GSDMD protein levels were increased (Fig. 4B and C). The most significant characteristic large vesicles from the plasma membrane in the SW620 cell line, and the most significant difference in expression in the N-terminal GSDMD protein, which occurred in the SW1463 cell line were selected for subsequent validation and detection of the molecular mechanism of pyroptosis induced by IL-17A treatment. We used scanning electron microscopy to observe the membrane morphology to determine whether IL-17A induces colorectal cancer cell pyroptosis. When colorectal cancer cells were stimulated with 100 ng/mL IL-17A for 72 h, numerous pores of varying sizes were distributed over the entire cell surface, and typical characteristics of pyroptosis, including cell swelling and large bubbles on the surface of the cell membrane, were observed (Fig. 4D). The release of lactate dehydrogenase (LDH) and IL-1β into the supernatant of cancer cells following stimulation was increased with IL-17A treatment (Fig. 4E and F). We used caspase-4/propidium iodide (PI) double staining to further identify and quantify pyroptosis. The caspase-4 + /PI + fluorescence intensity was significantly higher in the IL-17A group (SW620 group P = 0.0025 < 0.01; SW1463 group P = 0.0118 < 0.05) than in the control groups at 72 h (Fig. 4H and G).

IL-1β is one of the major inflammatory factors released during pyroptosis, therefore, we analysed the relationship between the expression of IL-17A and IL-1β in our cohort of 78 CRC cases. Pearson's correlation analysis indicated a significant correlation between the expression of IL-17A and IL-1β (r = 0.8895, P < 0.0001) (Fig 4I and J).

### IL-17A has direct effects on mitochondrial dysfunction

**Fig. 5:**
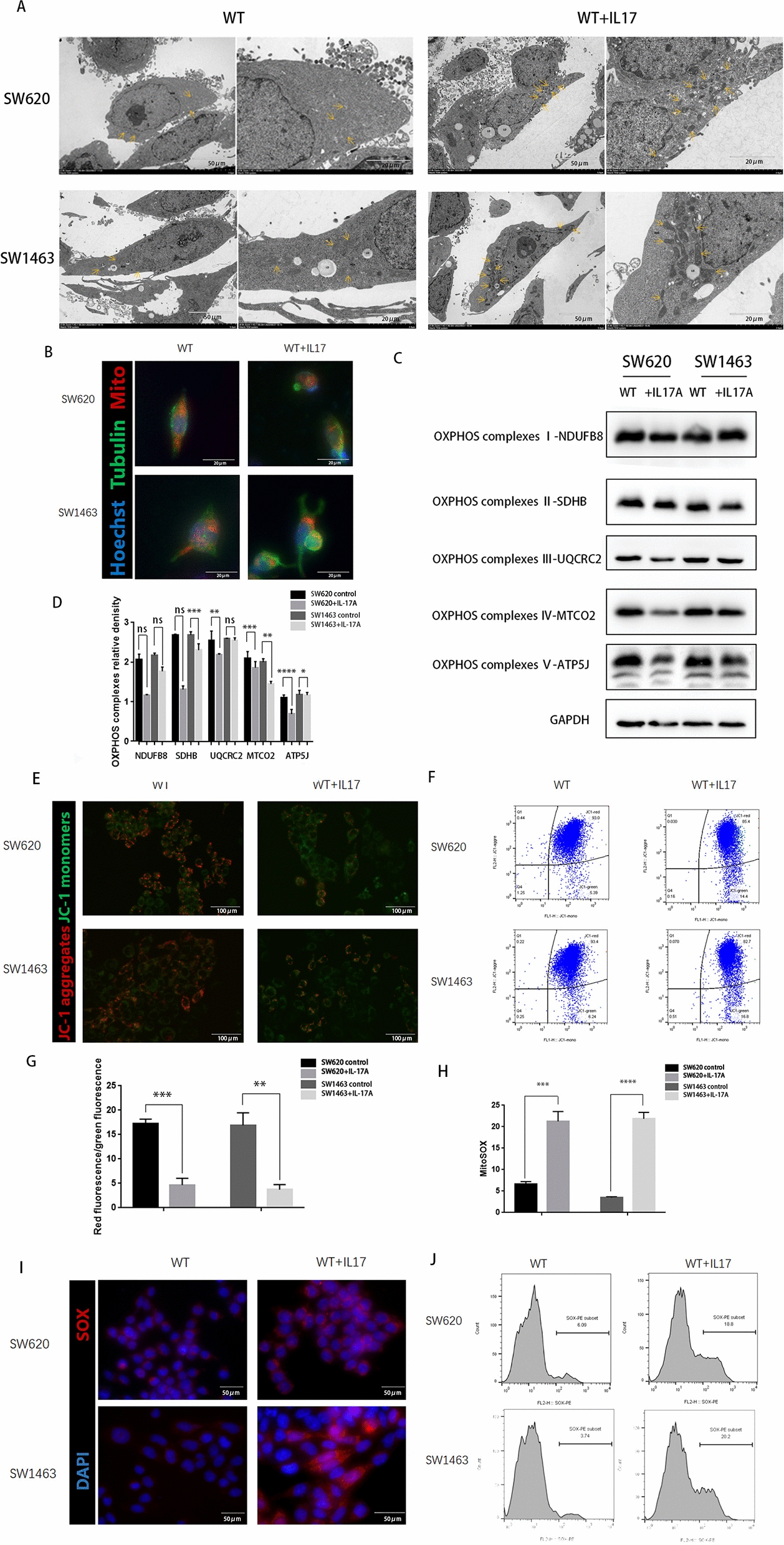
Mitochondrial dysfunction increases in CRC cells by treatment with IL-17A. **A** Transmission electron microscopy of CRC cells after treatment with IL-17A.Arrowheads indicate mitochondria; **B** CRC cells after treatment with IL-17A were immunostained with Mito-Tracker (red), anti-α-tubulin (green), and DAPI (nuclei, blue); **C**, **D** The expression of OxPhos complex subunits in CRC cells after treatment with IL-17A was analysed by western blotting, data conforms to normal distribution, and tested by two independent samples t-test. The mitochondrial membrane potential was measured using JC-1 dye. **E** representative microscopic and (**F**-**G**) flow cytometry analysis and quantitation, data conforms to normal distribution, and tested by two independent samples t-test. Immunostained for the Mitochondrial Superoxide Indicator with Mito-SOX. **H** representative microscopic and **I-J** flow cytometry analysis and quantitation, data conforms to normal distribution, and tested by two independent samples t-test

The results of transmission electron microscopy ultrastructural analysis revealed that in IL-17A-treated cells, the mitochondrial cristae collaped or even formed spots, whereas the morphology of the mitochondria in untreated cells did not show similar abnormalities (Fig. 5A). Mitochondrial dysfunction resulted in mitochondrial perinuclear clustering. MitoTracker staining showed that the mitochondria approached the nucleus after treatment with IL-17A (Fig. 5B). Next, we further investigated whether IL-17A could alter the composition or level of the mitochondrial respiratory machinery. Western blot results suggested that IL-17A decreases the expression of components of the OxPhos complex, which may lead to an impaired respiratory capacity of the mitochondria in colorectal cancer cells (Fig. 5C and D).

Mitochondrial dysfunction may dissipate the mitochondrial membrane potential (ΔΨm) [14] and lead to an increase in the level of reactive oxygen species (ROS) [15]. The results of JC-1 staining determined by confocal microscopy and quantitative analysis by flow cytometry showed that the ratio of green to red staining was significantly increased in IL17A-treated cells compared to untreated cells (Fig. 5E–G), which indicated a lower mitochondrial membrane potential and that IL-17A decreased the mitochondrial membrane potential. To confirm this result, MitoSOX staining was observed by confocal microscopy, and quantitative analysis was performed by flow cytometry, the results revealed that IL17A-induced mitochondrial disorder significantly the increased intracellular levels of mitochondrial ROS (Fig. 5H–J). These results imply that mitochondrial dysfunction is involved in IL17A-mediated pyroptosis in colorectal cancer cells.

### IL-17A upregulates NF-κB activation and induces mitochondrial dysfunction-activated inflammasomes to trigger caspase-4/GSDMD-induced pyroptosis

**Fig. 6:**
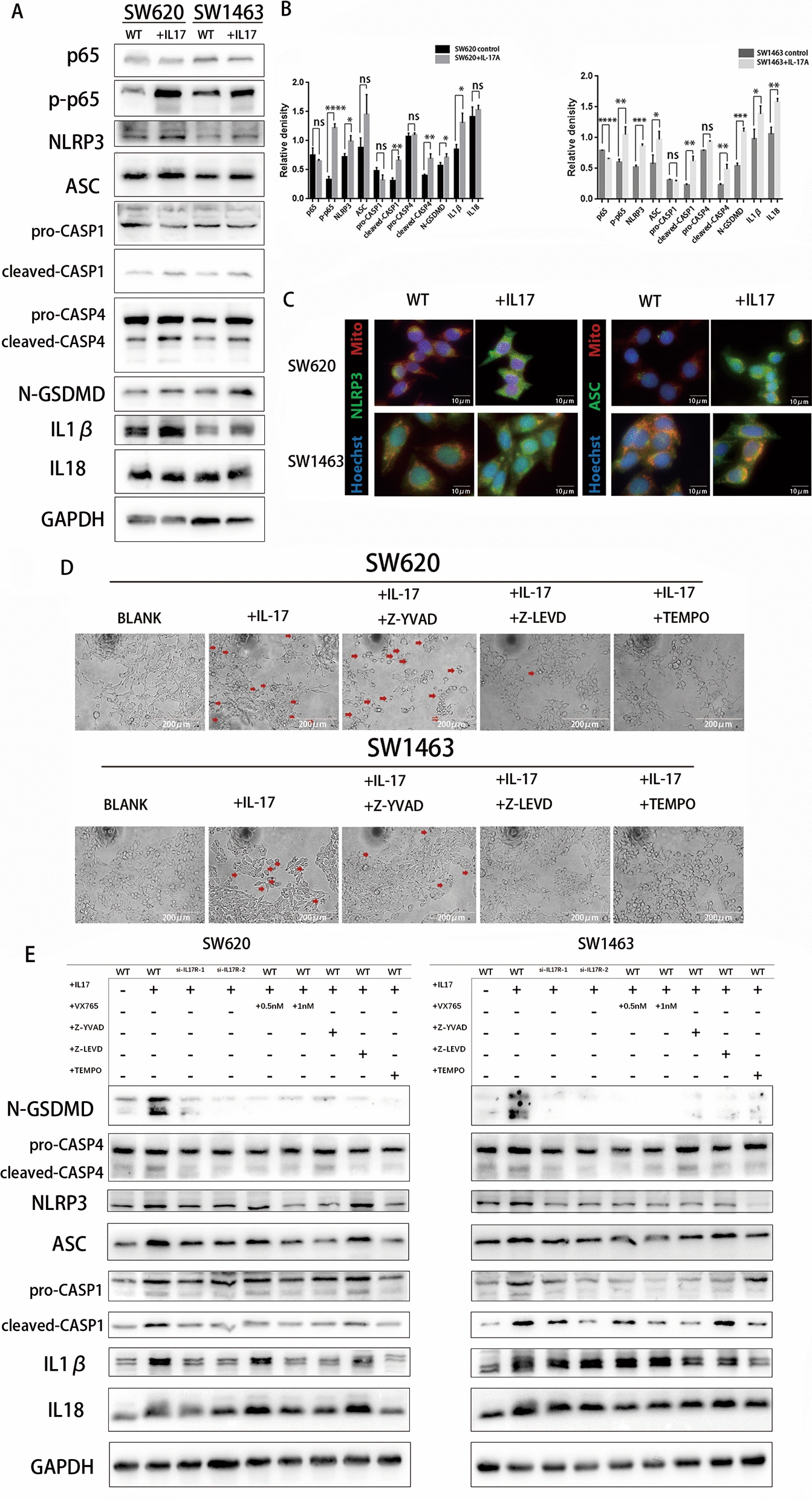
NF-κB activated pyroptosis-associated proteins treatment with IL-17A. **A**-**B** Protein levels of NF-κB p65, caspase 1, caspase 4, NLRP3, ASC, IL-1β, IL-18 and and GSDMD-N in CRC cells treated with IL-17A (100 ng/ml) for 72 h, data conforms to normal distribution, and tested by two independent samples t-test; **C** Representative IF staining of NLRP3 and ASC in CRC cells treated with IL-17A (100 ng/ml) for 72 h;** D** Representative microscopic images of CRC cells treated with IL-17A (100 ng/ml) and Z-YVAD(50 μM) or Z-LEVD(50 μM) or TEMPO(10 μM) for 72 h. Red arrows indicate the characteristic balloon in the cell membrane; **E** Protein levels of NF-κB p65, caspase1, caspase4, NLRP3, ASC, IL-1β, IL-18 and and GSDMD-N in CRC cells treated with IL-17A (100 ng/ml) and different types of inhibitors for 72 h

According to the KEGG enrichment pathway results and previous reports, the IL-17 family interacts with its corresponding receptors to activate downstream pathways, such as NF-κB signalling, to induce the secretion of many proinflammatory mediators [16, 17]. NF-κB activation and high expression of IL-1β are key components of pyroptosis [18]. Inspired by these findings, we confirmed that IL-17A can activate NF-κB by Western blotting, and the results also showed that caspase-4, caspase-1, and GSDMD were activated after treatment with IL-17A, and that the expression of the active forms of these proteins (cleaved caspase-4 and -1, N-terminal GSDMD) was significantly increased. Not only was there significant increase in the expression of the pyroptosis-related protein NLRP3, apoptosis-associated speck-like protein containing a caspase-recruitment domain (ASC), but also the expression of proinflammatory cytokines downstream of the NLRP3 inflammasome, namely, IL-1b and IL-18, was significantly increased (Fig. 6A and B). The same results were observed by IF staining (Fig. 6C).

To further identify the decisive players in pyroptosis, Z-YVAD, a specific inhibitor of caspase-1, and Z-LEVD, a specific inhibitor of caspase-4, were used. Moreover, Z-LEVD, but not Z-YVAD, significantly reversed the induction of pyroptosis in colorectal cancer cells by IL-17A treatment (Fig. 6D). To further confirm the effects of the pathway on IL-17A–induced colorectal cancer cell pyroptosis, we selected the two targets with the highest inhibition efficiency, si-IL17RA (Additional file 1: Fig.S2) and a caspase inhibitor (Z-YVAD, a specific inhibitor of caspase-1; Z-LEVD, a specific inhibitor of caspase-4; and VX-765, low-dose caspase-4 inhibition at a low dose, caspase-4 and caspase-1 inhibition at a high dose), as well as the mitochondria superoxide inhibitor Mito-TEMPO to analyse the pathway of IL-17A-induced pyroptosis (Fig. 6E).

As expected, the pyroptotic inhibitor VX765, Z-YVAD, Z-LEVD, and the mitochondrial superoxide inhibitor TEMPO all, reversed the effect of IL-17A on the expression of N-terminal GSDMD and the NLRP3 inflammasome. Furthermore, the caspase-4 inhibitor Z-LEVD and low-dose VX765 inhibited only the IL-17A stimulation-induced upregulation of cleaved caspase-4 and N-terminal GSDMD; they showed no significant effect on NLRP3 inflammasome (NLRP3, ASC, caspase-1) expression. In conclusion, IL-17A-induced pyroptosis is the result of the combined involvement of activated mitochondrial dysfunction, caspase-1 and caspase-4.

### IL-17A promotes CD8 + T-cell tumour infiltration of in vivo

**Fig. 7:**
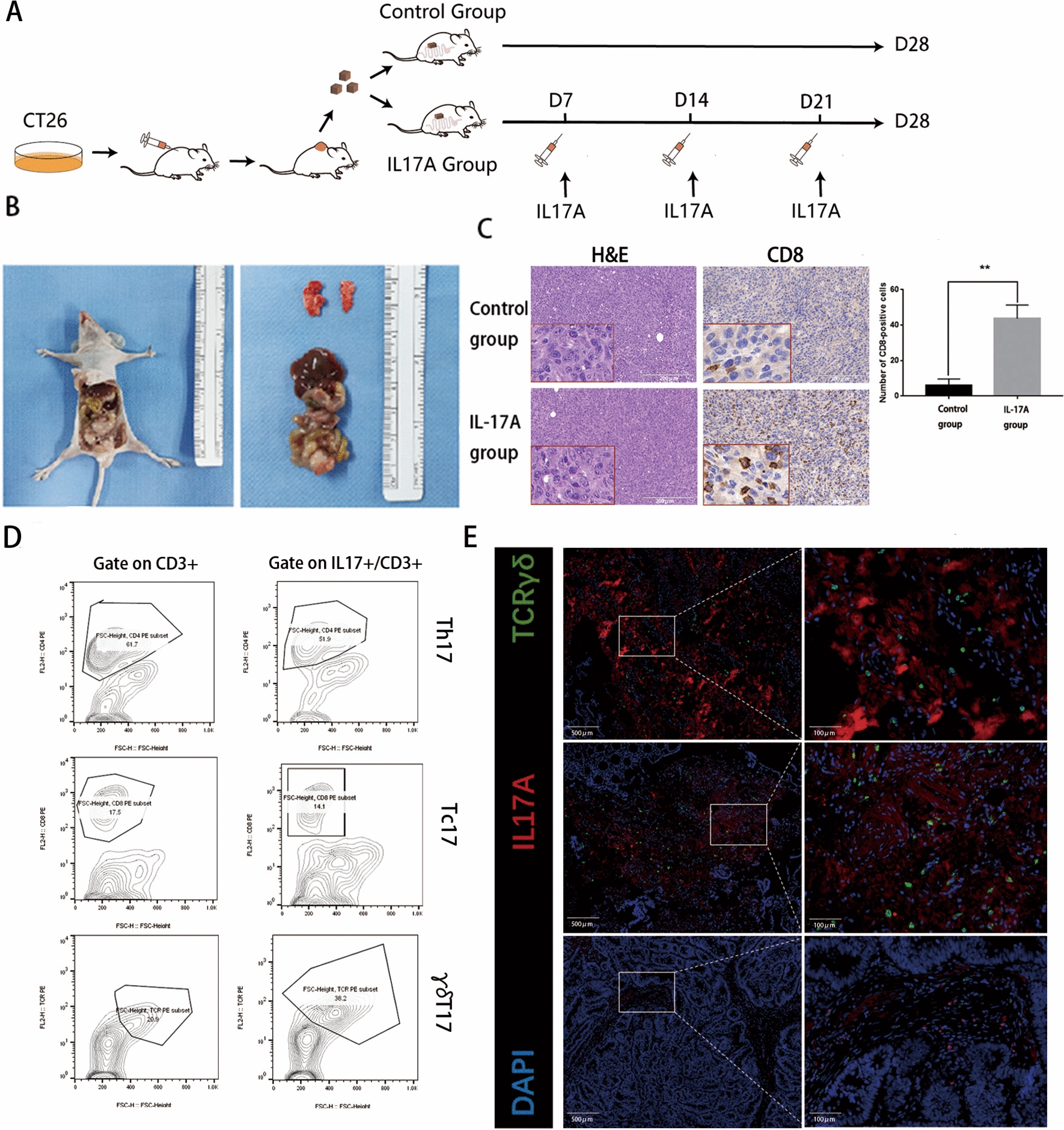
IL-17A are mainly producing by γδT cells and promotes CD8 + T cells infiltrating the tumour **A**-**B** The process of mouse-derived allograft colon cancer models; **C** H&E staining and immunostaining of CD8 from IL-17A or vehicle-treated colon tumour tissue. 3 fields under 40 × magnification were randomly selected, then counting and statistics CD8-positive cells, data conforms to normal distribution, and tested by two independent samples t-test; **D** Left panel representative flow cytometric analysis of all CD3 + cells from CD8 + T cells (Tc, top), CD4 + T cells (Th, middle), and γδTCR + T cells (γδT, bottom) and the right panel representative flow cytometric analysis of all CD3 + cells and IL-17A secretion from CD8 + T cells (Tc17, top), CD4 + T cells (Th17, middle), and γδTCR + T cells (γδT17, bottom) in tumour; **E** Paraffin sections from CRC patients were stained with anti-human pan-γδTCR (green) and anti-human IL-17A (red) for immunofluorescent (IF) staining

We established mouse-derived allograft colon cancer models by inoculating subcutaneous tumour tissue composed of CT26 cells into the mouse caecum mesenteric triangle (Fig. 7A and B). We performed immunohistochemical staining of tumours and showed that the proportion of CD8 + T cells was significantly increased in the mice in the IL-17A-treated group (Fig. 7C).

### γδT cells are the predominant IL-17-producing cells in human CRC

Previous studies have shown that IL-17 in colon tumours is produced by CD8 + T cells (Tc17), CD4 + T cells (Th17) and γδTCR + T cells (γδT17) [19]. To further investigate the source of IL-17A, we compared the proportions of CD8 + T cells, CD4 + T cells and γδTCR + T cells in CD3 + T cells in colon tumours, and the results showed that the proportion of γδTCR + T cells in IL17A + /CD3 + T cells was significantly increased compared to that in CD3 + T cells, while the proportions of CD8 + T cells and CD4 + T cells did not change (Fig. 7D). The IF staining results showed that γδT cells were readily observed in tumour with high IL-17A expression (Fig. 7E).

## Discussion

Previous studies have demonstrated that IL-17A plays an important role in tumour-associated inflammation and cancer development [20]. Despite substantial evidence suggesting that IL-17 family members are involved in colorectal cancer development [21], IL-17A expression correlates with a better prognosis and improved patient survival in colorectal cancer. The exact mechanism by which IL-17A engagement affects the tumour immune microenvironment to influence tumour progression has not been identified. In this study, we found a possible mechanism by which IL-17A regulates pyroptosis in colorectal cancer cells and mitochondrial dysfunction. Our data suggest that IL-17A-mediated mitochondrial dysfunction activates pyroptosis in colorectal cancer cells and promotes CD8 + T-cell infiltration into the tumour, which could be the key cause of the prognostic and regulatory potential.

The goal of this study was to investigate the cascade response induced in tumour cells in response to IL-17A in human colorectal cancer. We found that the upregulated DEGs were highly associated with nuclear transcription, and inflammatory factor production and secretion, and numerous of the activated pathways were associated with NF-kB, which was also confirmed in our subsequent experiments. By observing the ultrastructure of IL17A-treated colorectal cancer cells, we found numerous pores or pits of varying sizes on the cell membrane, structural collapse, flattening of the cell shape, disruption of the mitochondrial cristae, and rupture of the plasma membrane after IL17A treatment. Based on our observations, treatment with IL-17A enhanced perinuclear aggregation of the mitochondria, decreased the membrane potential, and increased the accumulation of intracellular ROS. Morphological and physiological disorders in the mitochondria reflect mitochondrial dysfunction and imply changes in the expression of components of the respiratory machinery [22]. IL-17A decreased the level of mitochondrial respiration and the protein levels of components of the respiratory chain complex. In our data, the influence of IL-17A was so significant as to disable a major mitochondrial function, and the destructive function-related accumulation of ROS resulted in the formation of inflammasomes.

Caspase-4 can be cleaved by NF-κB to participate in pyroptosis, which is considered a unique form of cell death in colorectal cancer cells [23]. In our study, both caspase-4 and caspase-1 were cleaved, and experimental data have suggested that caspase-4 can activate inflammatory caspase-1 in different inflammasome complexes and in different cell types [24]. To further clarify the function of the caspase-4, caspase-1 and ROS interaction induced by treatment with IL17A, we introduced competitive inhibitors of caspase-4, caspase-1 and ROS. The effects of Z-LEVD, Z-YVAD, VX765 and TEMPO were all attenuative, further demonstrating that all the factors were involved in the pathway of IL-17-mediated pyroptosis. Especially when the inhibitors of caspase-4 and ROS were used, IL-17 treatment significantly promoted the cleavage of GSDMD, which was entirely reversed by either Z-LEVD or high-dose VX765 and Mito-TEMPO. Blocking caspase-1 activation only partially inhibited GSDMD-mediated cell pyroptosis but completely suppressed activation of the NLRP3 inflammasome. Therefore, we proposethat IL-17A activates the NF-κB pathway, triggering the activation of caspase-4 and the cleavage of GSDMD in colorectal cancer cells. Moreover, IL-17A-mediated mitochondrial dysfunction induces ROS-induced activation of the NLRP3 inflammasome and cleavage of caspase-1, driving pyroptosis and eventually promoting CD8 + T-cell infiltration into the tumour microenvironment.

The novel observation of this work is that IL17-A promotes intracellular ROS accumulation by inducing mitochondrial dysfunction while inducing pyroptosis by activating the ROS/NLRP3/capsase4/GSDMD pathway, upregulating the release of inflammatory factors such as IL-1β, IL-18 and immune antigens into the tumour microenvironment, and recruiting CD8 + T cells to infiltrate the tumour in colorectal cancer.

Numerous studies have revealed that pyroptosis-related genes (PRGs) in CRC demonstrated their potential roles in the tumour-immune-stromal microenvironment, clinicopathological features, and prognosis [25, 26]. Pyroptosis in tumour cells would enhance the tumouricidal effect, various chemotherapeutic drugs can kill colorectal cancer cells via canonical or non-canonical pyroptosis [27, 28]. Our study found that IL-17A signalling can induce inflammatory death of colorectal cancer cells, releasing considerable immune antigens through cell pyroptosis and consequently recruiting CD8 + T-cell to activate tumour immunity. The immunomodulatory potential of IL-17A provides an attractive target for cancer immunotherapy. However, Gao Tan’s [29] study found that HMGB1, a proinflammatory factor released from GSDME-mediated pyroptotic epithelial cells, induces colorectal cancer proliferation through the ERK1/2 pathway. The effects of other intercellular substances in the tumour microenvironment, such as ATP, HMGB1, IL1β, and LDH, are still uncertain. This is a topic that deserves further study. From these results, we can see that pyroptosis mediates the tumouricidal effect of some inhibitors or cytolytic immune cells, and pyroptosis-released inflammatory factors may fuel the antitumour immunity or be harmful to patients by promoting tumour or causing inflammatory cascades. Thus, the relationship between pyroptosis and antitumour immunity is not certain and is worthy of further investigation.

However, there were some limitations to this study. We focused on pyroptosis mediated by IL-17A in colorectal cancer cells but did not further explore the exact mechanisms by which pyroptosis influences tumour-infiltrating T cells in the tumour immune microenvironment. Future studies might elucidate more comprehensive mechanisms underlying the regulation of tumour development and progression in colorectal cancer under conditions of IL-17A treatment.

## Conclusion

In this study, we demonstrate that IL-17A can induce mitochondrial dysfunction, stimulate intracellular ROS production and promote pyroptosis by activating the ROS/NLRP3/capsase4/GSDMD pathway, can recruits CD8 + T cells infiltrating to the tumour in colorectal cancer cells. The immunomodulatory potential of IL-17A provides an attractive target for cancer immunotherapy. In practical application, the paradoxical role of IL-17A in tumour growth and elimination makes a poor match with the "one-size-fits-all" approach. We considered that IL-17A was an important bridge between inflammation and immunity in colorectal cancer, the relationship between IL-17A and antitumour immunity is worthy of further investigation.

## Supplementary Information

**Additional file 1.** Fig.S1.

**Additional file 2.** Fig.S2.

## Data Availability

All data generated or analysed during this study are included in this published article.
