# Supplementary figures and images for: IL-17A-mediated mitochondrial dysfunction induces pyroptosis in colorectal cancer cells and promotes CD8 + T-cell tumour infiltration
Source: J Transl Med. 2023 May 21;21:335. doi: 10.1186/s12967-023-04187-3 (PMC10200054; doi:10.1186/s12967-023-04187-3)

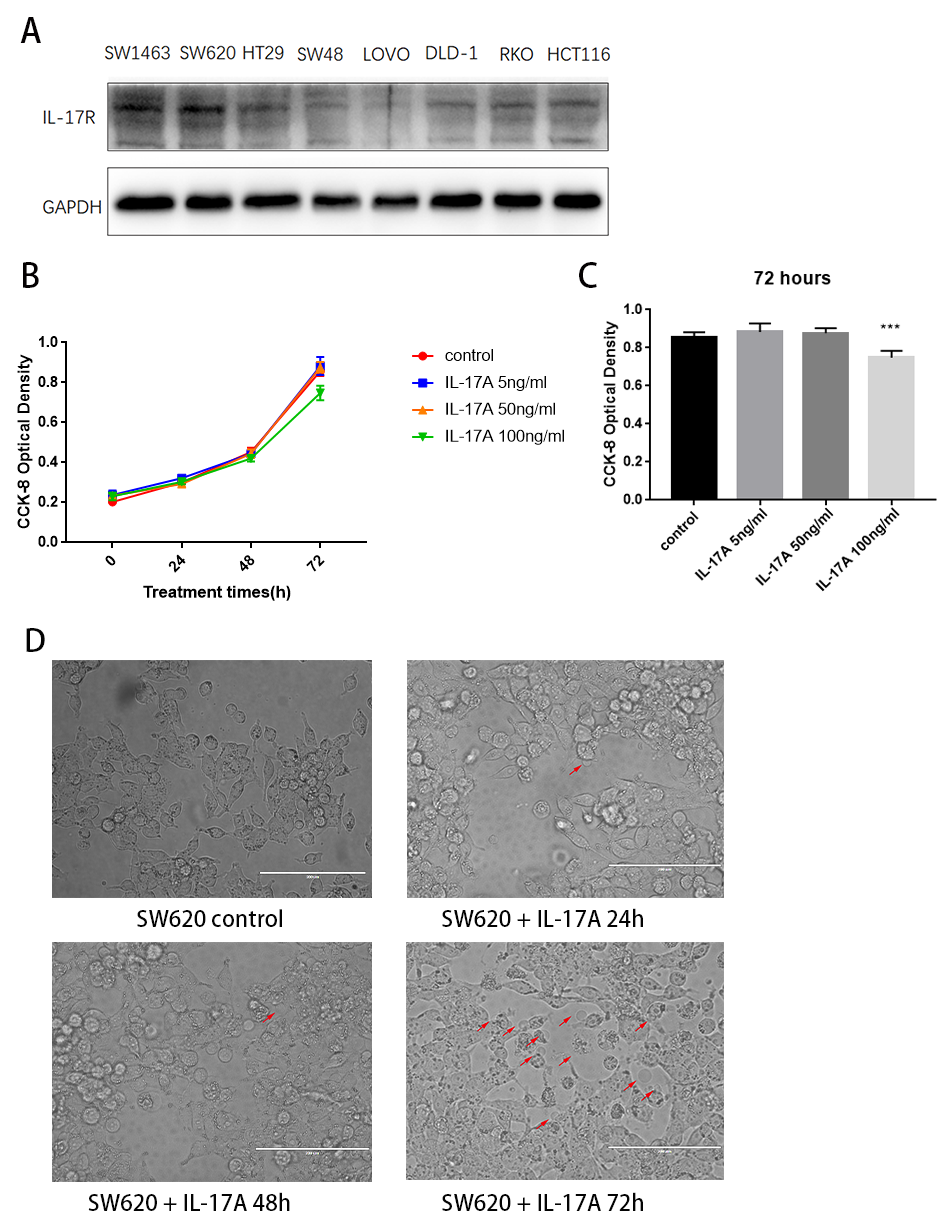

Supplement: Supplementary file 1 — Additional file 1. Fig.S1. [file 12967_2023_4187_MOESM1_ESM.tif]

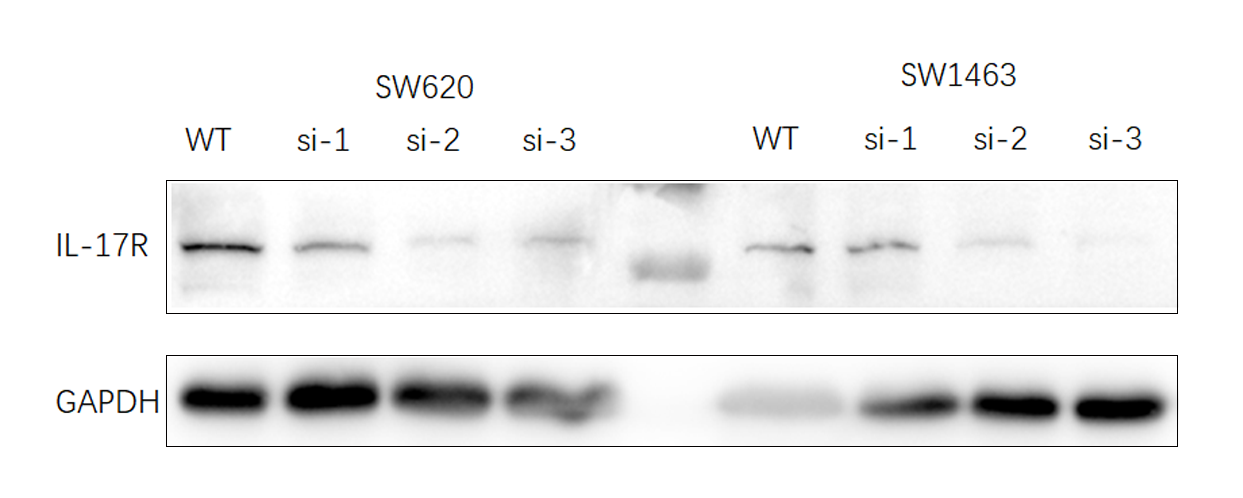

Supplement: Supplementary file 2 — Additional file 2. Fig.S2. [file 12967_2023_4187_MOESM2_ESM.tif]
